# MXene Enhanced the Electromechanical Performance of a Nafion-Based Actuator

**DOI:** 10.3390/ma15082833

**Published:** 2022-04-12

**Authors:** Xiaoming Tang, Ziyi Zhou, Yuehang Jiang, Qian Wang, Qi Sun, Lei Zu, Xing Gao, Huiqin Lian, Minhua Cao, Xiuguo Cui

**Affiliations:** 1 Beijing Key Lab of Special Elastomer Composite Materials, College of New Materials and Chemical Engineering, Beijing Institute of Petrochemical Technology, Beijing 102617, China; 2019540007@bipt.edu.cn (X.T.); zhouziyi@bipt.edu.cn (Z.Z.); jiangyuehang@bipt.edu.cn (Y.J.); wangqian@bipt.edu.cn (Q.W.); sunqi@bipt.edu.cn (Q.S.); lianhuiqin@bipt.edu.cn (H.L.); cuixiuguo@bipt.edu.cn (X.C.); 2 State Key Laboratory of Organic Inorganic Composites, College of Materials Science and Engineering, Beijing University of Chemical Technology, Beijing 100029, China; 3 School of Chemistry and Chemical Engineering, Beijing Institute of Technology, Beijing 100081, China; caomh@bit.edu.cn

**Keywords:** MXene, carbon nanotubes, actuation, back-relaxation, ionic electroactive

## Abstract

Ionic electroactive polymer-based actuators have attracted much attention due to their low potential stimuli. In this work, MXene–Nafion composite actuators were fabricated, and the actuation performances were tested. The morphology of the as-made MXene–Nafion composite showed that the composite membrane was homogeneous, with an MXene doping level up to 5 wt%. In addition, the results of blocked force, response speed, and durability demonstrated that the actuation behavior of the composite-based actuator was enhanced due to the efficient dispersion of the two-dimensional nanofiller MXene. In addition, the blocking force of the composite actuator with a doping level of 0.5 wt% was about 6 times that of the pure Nafion without back-relaxation and durability degradation during the testing period.

## 1. Introduction 

Ionic electroactive polymers (IEPs) have been attracting ever-growing attention owing to their virtues of good flexibility, high force density, and large displacement under low-potential (1–5 V) stimuli, which give them broad applicability in the field of flexible robots. Typically, IEP-based actuators possess a sandwich structure, i.e., an ionic polymer membrane sandwiched between two electrodes [1].

**Figure 1:**
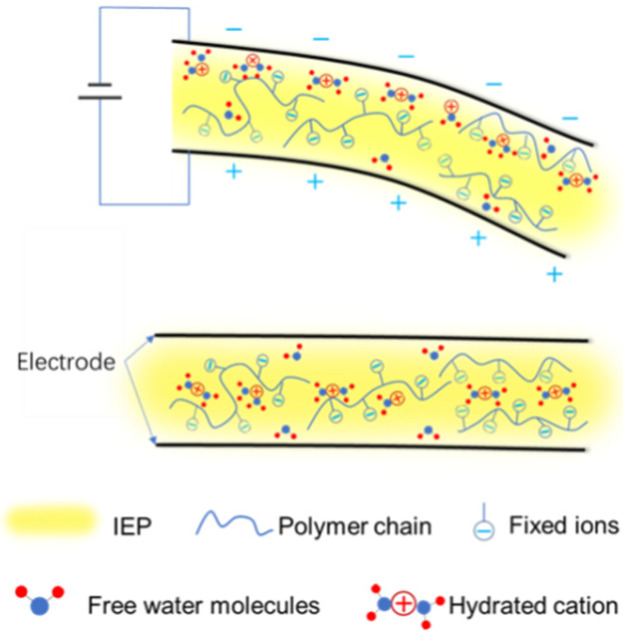
Schematic diagram of IPMC structure and actuation.

The electromechanical mechanism of IEP-based actuators consists of hydrated metal cations inside the actuator migrating toward the cathode under an electrical field, causing a large deformation and producing a strong blocking force, as shown in Figure 1. The electromagnetic performance of the actuator is determined by the amount of electrical energy is stored in a double interface, as well as the reversible migration of ion intercalation and de-intercalation at the interface of the electrode and the electrolyte membrane and in the electrode [2].

However, IEP-based actuators usually suffer from low generated force, back-relaxation under the DC field, and low durability, limiting their application [3,4].

Recently, Ma et al. [5] reported a Nafion-based actuator exhibiting a large tip displacement (35.3 mm) in the DC electric field and ultrafast response (>10 Hz) to the AC electric field that could be used in the field of biomic devices. Umrao et al. [6] reported that Nafion-based actuators with an ionically cross-linked Ti_3_C_2_T_x_ electrode showed high bending strains of 1.37% and ultrafast actuation response within 1 s without back-relaxation under DC input signals. All of this work indicated fast response and high deformation; however, the improvements in generated force and back-relaxation were limited.

Usually, two-dimensional (2D) nanomaterials with high specific surface area exhibit good adsorption and gas barrier properties. Therefore, they are ideal candidates as fillers in composites for hindering solvent evaporation [7,8]. Furthermore, our previous work showed that graphene could improve the generated force by 3 times comparing with that of pure Nafion [9].

It is well known that new 2D materials under the broad title MXene are very promising candidates for energy storage devices, sensors, and actuators. Among more than 20 different types of MXene, Ti_3_C_2_T_x_ exhibits excellent mechanical properties [10,11], electric conductivity [12], and biocompatibility [13,14], which makes it of possible interest for fabricating new-generation e-skin sensors [15]. Furthermore, some works have demonstrated that Ti_3_C_2_T_x_ could enhance the properties of polymers as a functional filler in terms of mechanical properties, heat resistance, sensitive properties, and capacitance [16,17].

In this work, we report an IEP actuator based on an MXene–Nafion composite with carbon nanotubes as electrode. MXene was used as functional filler to enhance the actuation performance of the actuators due to the interaction between the Nafion matrix and Ti_3_C_2_T_x_. The MXene nanosheets were homogeneously dispersed in the Nafion matrix with a doping level of 0.5–5 wt%. Nafion chains ionically bonded with Ti_3_C_2_T_x_ provide a synergistically favorable architecture for ion intercalation/de-intercalation, resulting in exceptionally high actuation performance compared with neat Nafion. The addition of MXene to the Nafion matrix improved the actuation behaviors of the IEP actuator, including blocked force, stress relaxation time, and durability, compared with those of the pure Nafion membrane. These results suggest that high-performance IEP actuators using a functional filler of MXene could be more suitable for practical engineering applications such as position control and precise force control.

## 2. Experimental Section

### 2.1. Materials

Nafion^®^117 solution (5 wt% in a mixture of 1-Propanol and Ethanol) was purchased from DuPont. Ti_3_AlC_2_ is from Brofosnano Technology Co., Ltd., Ningbo, China, *N*,*N*-Dimethyl formamide (DMF), multi-walled carbon nanotube (CNT), lithium fluoride (LiF) and hydrochloric acid (HCl) were obtained from Aladdin Co., Ltd., Shanghai, China.

### 2.2. The Preparation of MXene

Ti_3_C_2_T_x_ (T=OH, O, F) was prepared by etching the MAX phase Ti_3_AlC_2_ through LiF and hydrochloric acid according to references [6,11,18,19]. Typically, 1.8 g LiF was dissolved in 20 mL 10M HCl in Teflon beaker, then 1 g Ti_3_AlC_2_ powder was added to the beaker slowly and stirred for 5 min at 0 °C, followed by stirring for another 12 h at 45 °C. Then the mixture was filtrated, rinsed with 1M HCl 3 times, and with deionized water several times until pH ≈ 6, and then the filter cake was sonicated in 50 mL water at 10 °C for 4 h in an argon atmosphere. Finally, the dispersion was centrifuged at 5000 rpm, and the Ti_3_C_2_T_x_ powder was obtained by freeze drying of the supernatant.

### 2.3. The Preparation of MXene–Nafion Composite Membranes

A certain amount of MXene powder was dispersed in 20 g Nafion suspension with 20 mL DMF. In MXene–Nafion composites, the contents of MXene were 0.5, 1.0, 2.0, and 5.0 wt%, respectively, based on neat Nafion, labled as 0.5, 1, 2, and 5% MXene. The dispersion was first sonicated for 30 min, then mechanically stirred at 65 °C for 24 h. The obtained mixture was poured into a Teflon mold in an oven at 80 °C for 24 h forming a film with a thickness about 0.2 mm.

### 2.4. The Preparation of CNT Electrode

The CNT electrode was fabricated as follows: first, the electrode DMF dispersion with CNT oxide and Nafion in a mass ratio of 1 to 3 was prepared, the solid content of dispersion was 30 wt%; second, the strip of composite membrane was dipped into the electrode dispersion; and finally, the membrane was pulled out and dried at 80 °C for 10 min. This process was repeated 3 times to ensure a dense and uniform CNT layer on the membrane. After that, Li^+^-IEP was obtained by ion-exchanging the H^+^-IEP in 1 mol/L LiCl solution at room temperature for 24 h.

### 2.5. Characterization

The as-made MXene and membrane were characterized by X-ray powder diffraction (XRD, Scintag PAD X diffractometer, Cu Kα source, operated at 40 kV and 40 mA). Samples were scanned at 2.4°/min between 2θ of 5–70°.

Scanning electron microscopy (SEM) was performed with FEI Inspect F50 and Hitachi SU4800.

Raman spectra were acquired from InVia Raman microscope (Renishaw Inc., Gloucestershire, UK) equipped with a 9 mW power, 532 nm laser transmitter, the resolution of which was 0.02 cm^−1^.

Water uptake (*w*%) was calculated using Equation (1).

(1) $$w\%=\frac{w_{1}-w_{0}}{w_{0}}\times 100\%$$

Swelling (*s*%) was calculated by Equation (2).

(2) $$s\%=\frac{l_{1}-l_{0}}{l_{0}}\times 100\%$$

*w*_1_, *l*_1_ and *w*_0_, *l*_0_ are the weight and length of the swollen and the dried samples, respectively. Composite film was first vacuum-dried at 80 °C for 24 h. Then the film was immersed in deionized water at ambient temperature for 24 h. Wiping off the water on the surface with filter paper, it was immediately weighed. The ion exchange capacity (IEC) was determined by drying the film at 60 °C under vacuum until the weight was constant and then placing it in 1 mol/L NaCl solution at 25 °C for 24 h followed by titration using a 0.005 mol/L NaOH solution with phenolphthalein as an indicator. The IEC (meq g^−1^) was calculated based on the dry weight of the film.

Characterization of the actuation performance was carried out using a homemade setup according to reference [8].

All the IEP samples were cut into 25 mm × 5 mm dimensions, including the 5 mm of the electrode contact area. One end of the IEP strip was fixed between two Ti electrodes. All the determinations were carried out in air at room temperature.

## 3. Results and Discussion

**Figure 2:**
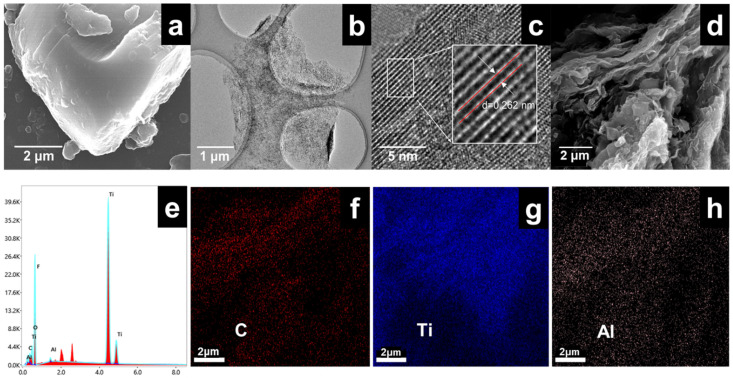
(**a**) SEM image of Ti_3_AlC_2_, (**b**) TEM image of MXene flake, (**c**) HR-TEM image of MXene, (**d**) SEM image of MXene film, (**e**) EDS spectrum, and (**f**–**h**) distribution diagram of C, Ti and Al elements corresponding to (**d**).

The morphologies of Ti_3_AlC_2_ and Ti_3_C_2_T_x_ MXene are shown in Figure 2. From the SEM images, Ti_3_AlC_2_ exhibits layers stacked tightly together without any cracking (Figure 2a). The TEM images reveal that the Ti_3_C_2_T_x_ nanosheets are highly exfoliated (Figure 2b), and the HRTEM image in Figure 2c shows the regular lattice fringes with *d* = 0.262 nm, corresponding to the (10–10) plane of Ti_3_C_2_Tx. Based on the values of *d*, the lattice constant *a* is about 0.303 nm, which is similar to that reported in the literature (lattice parameter *d* = 0.265 nm, lattice constant *a* = 0.307 nm) [20,21]. The EDS spectrum (Figure 2e–h) demonstrates C, Ti and Al elements in MXene corresponding to Figure 2d, and the atomic% of Al elements are 0.5% (among the C, Ti, Al, O, F), which verified that the Al element in the prepared MXene was almost etched. The atomic% of O and F in the prepared MXene samples are 15.6% and 47.6%, respectively. The presence of OH, O or F contributes to the bonding of Nafion with the MXene.

**Figure 3:**
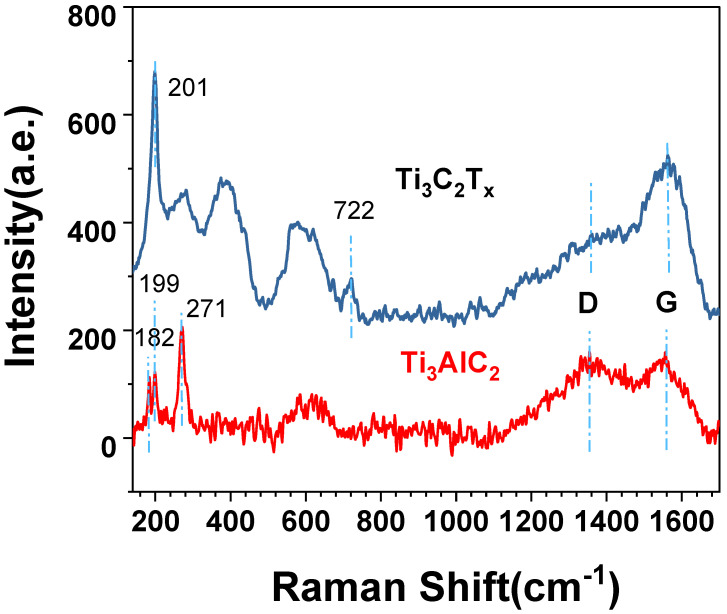
Raman spectra of Ti_3_C_2_T_x_ and Ti_3_AlC_2_.

In the Raman spectrum of Figure 3, MXene displays two peaks, of D-band around 1350 cm^−1^ and G-band around 1560 cm^−1^, which are typical carbon peaks [22]. D-band is a peak generated by disordered carbon from the defects in carbon-based materials [23], while the G band is a characteristic peak of the crystalline state of graphite due to the vibration of sp^2^-bonded carbon atoms in a two-dimensional hexagonal lattice [7]. It appears clearly that the intensity of the D-band is lower after etching, pointing out a reduction of disorder carbon, which may be due to the exposure of graphite and the removal of disordered carbon that does not enter the crystal in the raw material during formation process of Ti_3_AlC_2_. The peaks at 182, 199 and 271 cm^−1^ are attributed to vibrations of the Al atoms, and the peaks around 640 cm^−1^ are due to the vibrations of the C atoms in Ti_3_AlC_2_ [24]. After etching treatment, the Al related vibration peaks are disappeared in MXene spectrum, confirming the Al layer was etched. The sharp peak of MXene at 201 cm^−1^ is attributed to A_1g_ (Ti, O, C) and the vibration peaks at 396 cm^−1^ and at 632 cm^−1^ correspond to the Ti_2_ and C atoms of Ti_3_C_2_O_2_ and Ti_3_C_2_(OH)_2_, respectively [25]. The peak around 720 cm^−1^ is due to A_1g_(C) [26].

**Figure 4:**
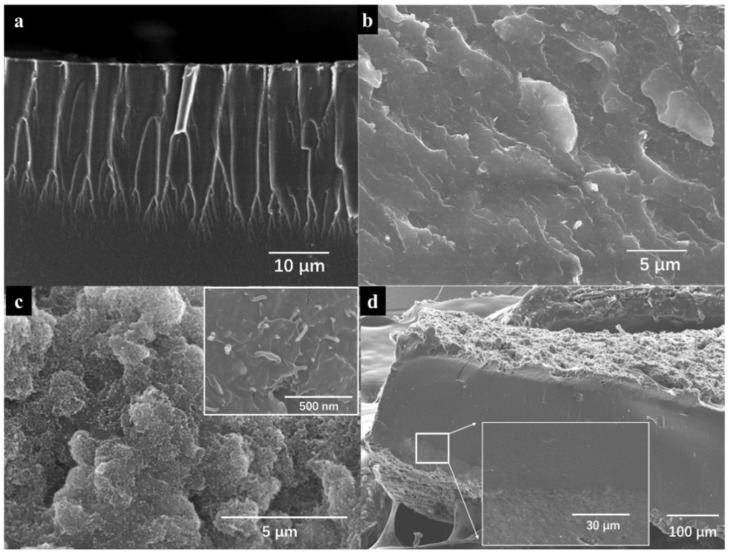
SEM images of (**a**) pure Nafion; (**b**) 2% MXene–Nafion composite film; (**c**) electrode horizontal surface of IEP; (**d**) neat Nafion film with CNT electrodes.

SEM images of the cross-section of the as-made pure Nafion and 2% MXene–Nafion composite membranes are shown in Figure 4. It can be observed that pure Nafion membrane appears to possess a smooth surface (Figure 4a), while MXene–Nafion composite membrane exhibits a rough surface, which may result from the interaction between the MXene and Nafion (Figure 4b). It has been reported [6] that a strong interaction force between MXene and sulfonate polymer is generated due to the noncovalent hydrogen bonding between −OH termination of Ti_3_C_2_T_x_ with −SO_3_H and SO_3_− group of polymer. Therefore, we believe that the noncovalent hydrogen bonding exists in MXene–Nafion composites.

On the basis of the SEM image of MXene–Nafion composite membrane (Figure 4b), the MXene is dispersed in the Nafion matrix homogenously. It can be observed that MXene parallels the surface of the film, maybe due to the gravitational forces experienced by MXene in the solution. The preferential orientation of MXene in the composite will benefit the durability of the actuator because of the gas barrier function of MXene.

In the IEP system, the morphology of the electrodes has an important impact on the electromechanical performance in terms of blocking force and response time. Using CNT as the electrode, the surface of IEP is uniform, as shown in Figure 4c,d. It can be seen that IEP presents a sandwich structure with an electrode layer thickness of about 45 μm. At the same time, the enlarged view shows that the electrode layer and the intermediate matrix layer are closely combined [27]. Based on the actuation mechanism of IEP, the electromechanical response of IEP is that the electric field acts on the matrix through the carbon electrode, and the perfect combination of the electrode layer and the base membrane is conducive to the migration of cations, resulting in larger actuation [28,29].

**Figure 5:**
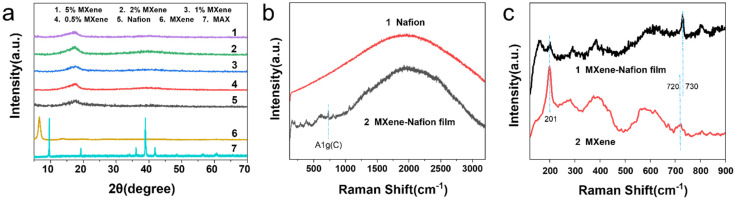
(**a**) XRD patterns of Ti_3_AlC_2_, neat Nafion, MXene–Nafion films; (**b**) Raman spectra of Nafion and MXene–Nafion film; and (**c**) Raman spectra of MXene and MXene–Nafion film.

Figure 5a shows the XRD patterns of the MAX phase Ti_3_AlC_2_ crystal, neat Nafion, and MXene–Nafion films. Compared with Ti_3_AlC_2_, MXene shows a broad peak (002) at 7.2° without other characteristic diffraction peaks of Ti_3_C_2_T_x_, revealing that the Al layers are completely removed after the etching process. It can be observed that, compared with Ti_3_AlC_2_, the stacking peak (002) of Ti_3_C_2_T_x_ is obviously shifted to a low angle and widened. This is because the crystal of the MXene phase expands along the c−axis during lamellar separation, which increases the lattice parameter c, resulting in a shift of the (002) peak to a low angle. The greater the offset, the greater the expansion degree of the c−axis of MXene crystal and the greater the distance between the layers [30].

On the basis of the XRD patterns of the as-prepared MXene–Nafion membranes, no peaks of MXene appear in any of the composite membranes, indicating that the MXene sheets are well dispersed in the Nafion substrate, without agglomeration or orientation behavior [8]. According to the literature, the peak around 17° present in all membranes is due to the Nafion phase. It can be decomposed into two peaks: a broad non-crystalline peak at 2*θ* = 16.7° and a sharp peak at around 2*θ* = 18° of crystalline regions of Nafion [31]. From Figure 5a, it can be seen that all of the composite peak is at 17.2° when the content of MXene is lower than 2 wt%. When it is 5 wt%, the peak of the composite shifts a little bit from 17.2° to 17.5°. Therefore, the addition of MXene does not influence the crystallization behavior of Nafion too much.

In Figure 5b, presenting the Raman spectra, pure Nafion film can be seen to exhibit a broad carbon peak. Meanwhile, in the composite film spectrum, the characteristic peaks of MXene are well maintained. As can be observed in Figure 5c, the A_1g_ (C) shifts to 730 cm^−1^ compared with raw MXene. Once the hydrophilic Nafion polymer is introduced, the interlayer spacing becomes larger, which exacerbates out-of-plane A_1g_ (C) vibrations peak [26]. The appearance of a peak at 156 cm^−1^ illustrates the formation of anatase caused by ablation with the Raman laser source [32].

The physical properties data with respect to term of swelling in water and ionic exchange capacity of pure Nafion and its MXene composite membranes are summarized in Table 1. The weight gain of pure Nafion in water is about 34.7%. Meanwhile, in the case of composite membranes, all the values are higher than that of pure Nafion. The sample of 2% MXene–Nafion possesses the highest water uptake of 52.1%. When the concentration of MXene was increased to 5 wt%, the weight gain of the MXene–Nafion composite membrane decreased to 37.0%. For the swelling behavior in water, the sample of 1% MXene–Nafion possesses the highest swelling with value of 20.4%, and when MXene loading was increased to 5 wt%, the minimum of the swelling of 11.8% was observed. The sample of 0.5% MXene–Nafion shows an IEC of 0.947 meq/g, which is the highest value among all of the samples.

It is well known that the presence of nanofillers can influence the size of polar clusters and the distribution of water domains in polar cages and in interconnection channels [9], which are responsible for both water uptake and IEC. The decrease in water uptake and the IEC of the high MXene concentration in composite membranes may be due to MXene decreasing the size of the ion clusters and the number of exchange sites in each cluster.

**Figure 6:**
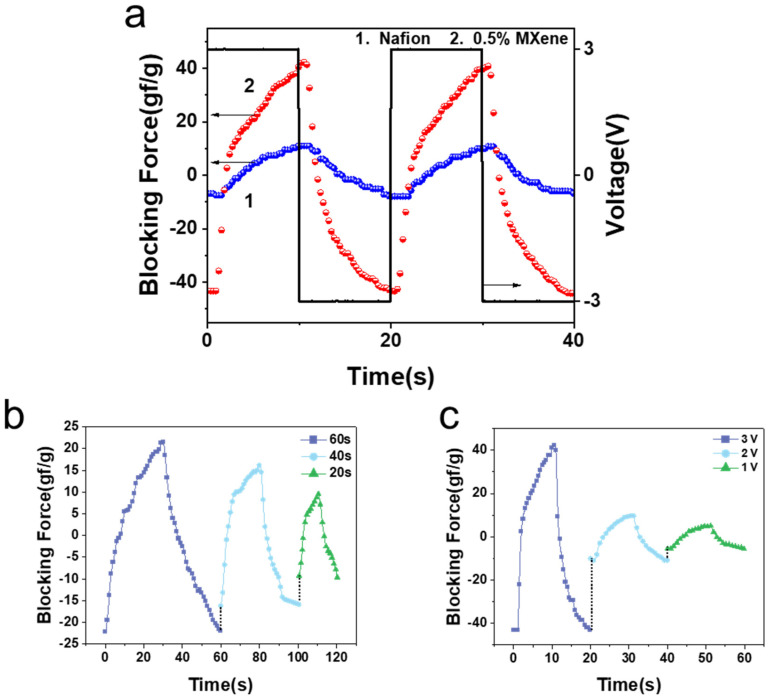
(**a**) Blocking force of MXene–Nafion actuators with period of 20 s. 1. pure Nafion; 2. 0.5% MXene–Nafion. (**b**) Blocking force of 0.5% MXene–Nafion actuators under ±2 V AC square wave. (**c**) Blocking force of 0.5% MXene–Nafion actuators with period of 20 s.

The blocking force is measured according to reference [8]. As can be seen in Figure 6, the 0.5% MXene–Nafion film generated the highest blocking force of 43 gf g^−1^ under 3 V, which is 4 times that of pure Nafion-based actuator of 10 gf g^−1^. Umrao reported that [6] Ti_3_C_2_T_x_ exhibits metal-like conductivity and high storage capacity, and the interlayer spacing of Ti_3_C_2_T_x_ benefits the intercalation and de-intercalation of Li^+^, thus improving the blocking force of IEP. The blocking force of the 0.5% MXene–Nafion actuators with different periods and voltages is shown in Figure 6b,c.

**Figure 7:**
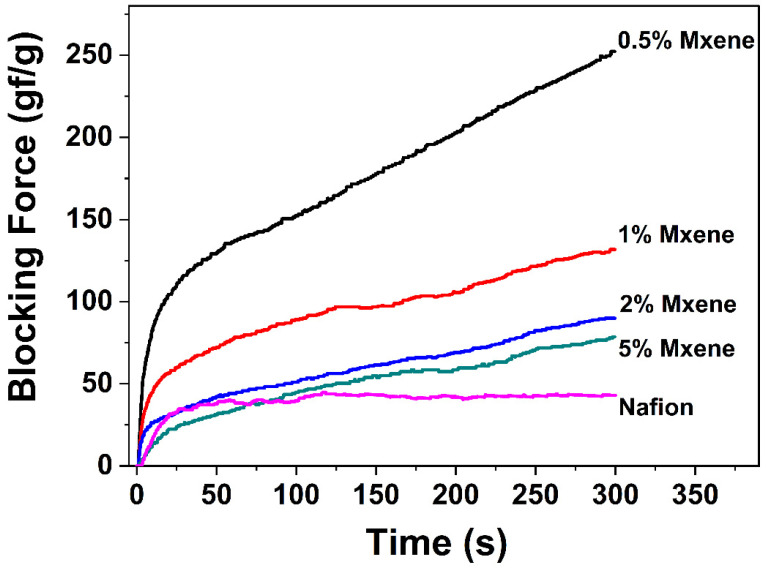
Variation of the blocking force with time of IEP films under 3V DC field.

According to the driving mechanism of IEP, the migration of hydrate cations causes deformation of the membrane, thus generating force. Under DC field, however, the hydrated cation in IEP shows reverse diffusion due to the repulsion between ions and the water penetration on the cathode side of the membrane, causing back-relaxation. Figure 7 shows the variation in the blocking force with time of the IEP films under a 3V DC field. For the pure Nafion-based IEP film, the blocking force first increased with time, reached its highest point of 44.8 gf g^−1^ at 117 s, and then the blocking force decreased with time. This is because the reverse diffusion of cations in Nafion-based IEP causes a recovery of driving force. The actuator with the MXene doping level of 0.5% showed the highest blocking force value among all actuators, of 252 gf g^−1^ at 300 s. Moreover, the back-relaxation phenomenon does not appear in MXene–Nafion composite-based actuators, and the curve showed a continuously increasing blocking force value throughout the whole testing period (300 s). In contrast with the pure Nafion actuator, MXene filling mitigates the back-relaxation effect of composite IEP, which makes the actuators more able to meet the requirements of precise control fields.

It can be observed that the driving force values of the composite IEP filmds decreased with increasing MXene content. This is because, according to the literature [33], the lamellar structure of MXene can attract lithium ions. Therefore, it is considered that MXene attracts lithium ions in this IEP system and limits the diffusion of cations, so it effectively increases the driving force and delays the arrival of backward relaxation [8,34]. However, with increasing MXene doping content, the driving force of IEP decreases. It can be inferred that MXene is a rigid material, which limits the large deformation of IEP. The sheet structure not only blocks the back infiltration, but also hinders the fast movement of hydrated ions. Therefore, the higher doping amount reduces the actuation ability.

**Figure 8:**
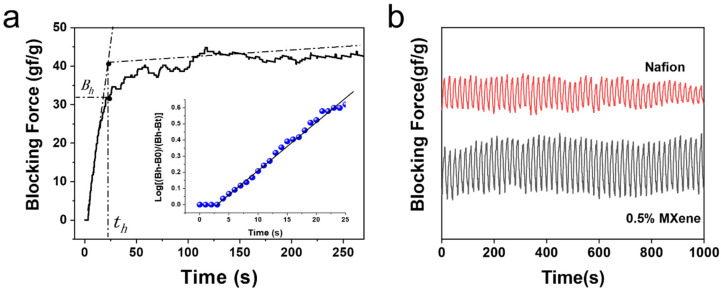
(**a**) Variation of the blocking force with time and the first-order plot (insert) of Nafion under 2.5 V field with period of 20 s; (**b**) durability of Nafion and 0.5% MXene sandwich film.

Figure 8a shows the relationship between *log*[(*B_h_* − *B*_0_)/(*B_h_* − *B_t_*)] and time, which was calculated on the basis of the data in Figure 7. I can be observed that at the initial stage, the response performance coincides with the first-order kinetics equation, i.e., *log*[(*B_h_* − *B*_0_)/(*B_h_* − *B_t_*)] = *k_t_*⋅*t*, where *B_h_* is the blocking force at the inflection point time (*t_h_*) under the test condition, as shown in Figure 8a, *B*_0_ is the initial value, and *B_t_* is the value at time *t*. *k* is the actuation rate constant. Detailed data of MXene–Nafion sandwich films are listed in Table 1. The fastest was the membrane of 2 wt% MXene (*k* = 0.252 s^−1^), while pure Nafion film showed the lowest value of *k*, at 0.027 s^−1^. We propose that conductive MXene fillers benefit the charge transport in composite, resulting the fast response speed. Simultaneously, the MXene layer blocked the movement of ions, thus resulting in a decrease in response speed with increasing MXene content.

Astonishingly, the highest blocking force generated by the 0.5% MXene–Nafion sandwich film under 3 V at 300s reached up to 252 gf g^−1^, nearly 6 times that achieved by pure Nafion (Figure 7), without any decrease with increasing time. For comparison, the neat Nafion actuator showed a gradual decrease in blocking force with increasing time. The mechanism of the strong enhancement blocking force of MXene deserves further investigation for a new generation of robust artificial muscle.

Figure 8b presents the durability of pure Nafion and 0.5% MXene–Nafion-based actuators. It can be seen that the blocking force (the highest value minus the lowest value in the cycles) of the pure Nafion-based actuator decreased with increasing time, while that of 0.5% MXene–Nafion-based actuator increased with increasing time. It can be observed that 2D nanofiller could improve the durability of the Nafion-based actuator due to the hindrance of water evaporation. It has been reported that Nafion-based actuators with a water electrolyte show a distinct electromechanical attenuation during testing due to the evaporation of water [35]. In our actuators, the lamellar structure of MXene protects the interlayer liquid from evaporation, thereby improving the durability of the actuators.

## 4. Conclusions

In conclusion, MXene was used as a new functional filler of ionic polymer-based actuator, resulting in obvious enhancement of its ambient durability. MXene disperses in Nafion suspension with different doping levels from 0.5 to 5 wt%. With MXene loading of 0.5 wt%, the composite actuator showed the best actuation behavior in terms of blocking force and durability. However, when the MXene content was higher than 0.5 wt%, the electromechanical performance decreased with increasing MXene content. These are all favorable for the further design and controlled preparation of the MXene–Nafion-based IEPs.

## Figures and Tables

**Table 1:** Physical properties of Nafion and Nafion−MXene composite membranes.

Film	Water Uptake (%)	Swelling (%)	IEC (meq/g)	*k* (s^−1^)
Pure Nafion	34.7	15.0	0.918	0.027
0.5% MXene	44.5	17.5	0.947	0.150
1% MXene	51.9	20.4	0.930	0.160
2% MXene	52.1	18.3	0.924	0.252
5% MXene	37.0	11.8	0.899	0.030
